# Robotic-assisted intracorporeal radical cystectomy with modified ileal conduit reduces ureteroileal anastomotic stenosis risk: a comparative study with laparoscopic surgery

**DOI:** 10.1080/07853890.2026.2703361

**Published:** 2026-07-29

**Authors:** Tengfei Gu, Roberta Gunelli, Ting Chen, Chen Wang, Yongtao Pan, Qinzhou Yu, Jie Li

**Affiliations:** a Department of Urology, Lishui Municiple Central Hospital, The Fifth Afliated Hospital of Wenzhou Medical University, Lishui, China; b Department of Urology, Morgagni-Pierantoni Hospital, Azienda USL della Romagna, Forlì, Italy

**Keywords:** Ileus, modified ileal conduit, robotic radical cystectomy, totally intracorporeal surgery, ureteroileal anastomotic stenosis, urinary diversion

## Abstract

**Background:**

This study aims to evaluate the clinical efficacy of an innovative robot-assisted totally intracorporeal antecolic modified ileal conduit procedure (robotic group) versus laparoscopic surgery (laparoscopic group) in bladder cancer patients, providing evidence for optimizing urinary diversion strategies and reducing associated complications.

**Methods:**

From Jan 2023 to Oct 2025, 69 patients undergoing total cystectomy by one surgeon were enrolled; 23 received robotic surgery (KangDuo SR2000) and 46 laparoscopic. Baseline, perioperative, and postoperative data were collected. Primary endpoint: radiologically confirmed ureteroileal anastomotic stenosis; secondary: postoperative ileus, new upper urinary tract stones, 90‑day complications, and recovery indicators.

**Results:**

Baseline characteristics were similar between groups (all *p* > 0.05). The robotic group had significantly lower stenosis (0% vs 15.22%, *p* = 0.043), ileus (8.70% vs 28.26%, *p* = 0.043), and new stones (4.35% vs 21.74%, *p* = 0.046). Intraoperative blood loss was reduced (98.70±28.97 vs 155.00±65.76 mL, *p* < 0.001), but operative time was longer (249.7±19.7 vs 199.6±22.7 min, *p* < 0.001). No significant differences were found in time to bowel function recovery or 90‑day complication rate.

**Conclusion:**

Robot‑assisted intracorporeal radical cystectomy with modified ileal conduit ensures perioperative safety, lowers long‑term complications and blood loss despite longer operative time, suggesting it is a promising alternative; however, prospective randomized controlled trials are needed to confirm these findings.

## Introduction

Bladder cancer ranks as one of the most common malignancies of the urinary system. For patients with muscle-invasive bladder cancer, high-risk non-muscle-invasive bladder cancer, or those refractory to conservative treatments, radical cystectomy remains the standard therapeutic approach [1]. As the predominant form of urinary diversion, the ileal conduit is widely utilized in clinical practice due to its relative technical simplicity and manageable complications [2]. However, the procedure still faces significant challenges, among which ureteroileal anastomotic stricture and postoperative ileus represent two major complications affecting long-term patient outcomes. Their reported incidence ranges from 4% to 16% [3], substantially impairing both quality of life and upper urinary tract function.

Currently, the most commonly employed surgical technique in clinical practice involves tunneling the left ureter posterior to the colon to bring it to the right side for ureteroileal anastomosis [4]. Despite its widespread use, studies have revealed significant anatomical drawbacks associated with this approach. The procedure necessitates extensive mobilization of the left ureter to allow its transposition through the retrocolic tunnel. This not only risks compromising the ureteral adventitial blood supply, potentially leading to ischemic stricture, but also predisposes the ureter to compression and angulation as it courses behind the colon, further increasing the risk of stenosis [5].

To overcome the limitations of the conventional technique, some surgeons have attempted to pass the ileal segment posterior to the colon to the left side for an end-to-end anastomosis with the left ureter [6]. This modified approach avoids the extensive mobilization of the left ureter, theoretically reducing the risks of ischemia and angulation. However, this procedure demands extensive mobilization of the ileal mesentery, carrying significant risks of serious complications such as ileal segment ischemia and mesenteric torsion. Furthermore, it often requires open surgery, which limits its widespread clinical application [7].

Against this backdrop, we innovatively performed a robot-assisted and fully intracorporeal modified ileal conduit procedure. The core technical innovation of this study lies in routing the ileal conduit anterior to the colon to the left side for an end-to-end anastomosis with the left ureter. This novel pathway design not only preserves the advantage of avoiding excessive ureteral mobilization and protecting its blood supply but also circumvents the risk of mesenteric torsion associated with the retrocolic route, offering a novel technical solution for reducing the incidence of ureteroileal anastomotic stricture and postoperative ileus.

Robot-assisted laparoscopic surgery is considered a major leap in minimally invasive surgery, which combines the dexterity of open surgery and the microinvasive technique of laparoscopic surgery. Despite its widespread adoption, the high cost remains the most prominent drawback of the da Vinci surgical system [8]. In response to this limitation, a variety of alternative robotic platforms have been developed across different countries, including the Revo-i system and the Senhance robotic platform. In China, the domestically engineered KangDuo surgical robot system has exhibited remarkable clinical efficacy in a range of procedures, such as partial nephrectomy, pyeloplasty and radical prostatectomy [9–11].

Currently, there is a lack of systematic evidence-based medical evidence regarding the clinical outcomes of this innovative approach. To address this gap, this study conducted a single-center analysis to systematically compare the clinical efficacy of the robot-assisted, fully intracorporeal, antecolic modified ileal conduit procedure with that of the traditional technique. The evaluation focused on the advantages of this innovative procedure in preventing ureteroileal anastomotic stricture and postoperative ileus, aiming to provide reliable evidence for the optimization of urinary diversion techniques in bladder cancer surgery.

## Materials and methods

### Patients

**Figure 1.**
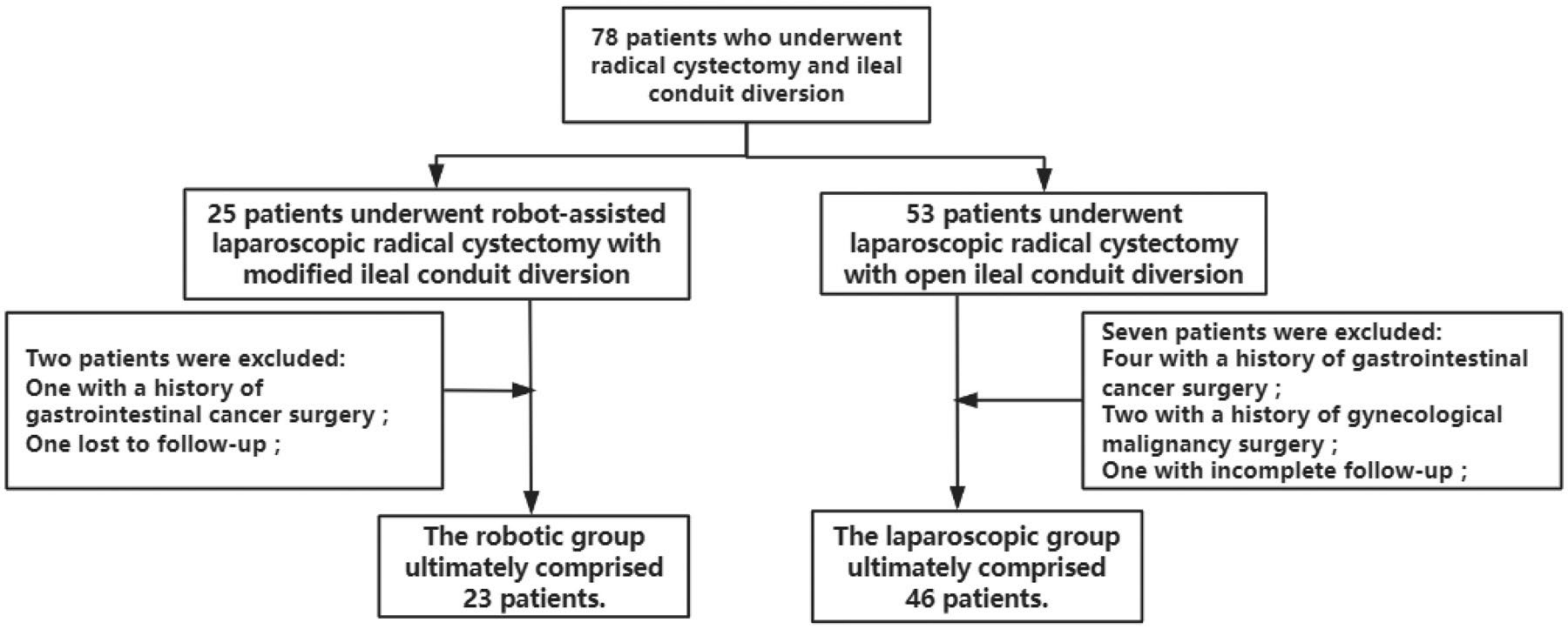
Flowchart of patient enrollment and exclusion in this study.

Patient data were extracted from the bladder cancer database of the Department of Urology at Lishui Central Hospital in Zhejiang, China. This study included all patients who underwent radical cystectomy for bladder cancer with ileal conduit diversion by the same surgeon. Patients receiving palliative cystectomy were excluded. From 1 January 2023 to 31 October 2025, 78 patients were enrolled. Subsequently, 5 patients were excluded due to severe abdominal adhesions from previous abdominal surgeries, and 4 patients were excluded due to incomplete follow-up. Ultimately, 23 patients underwent robotic-assisted intracorporeal radical cystectomy with a modified ileal conduit, while 46 patients underwent the laparoscopic approach (Figure 1). During the study period, no patients at our institution received neoadjuvant chemotherapy. Patients who received neoadjuvant chemotherapy were excluded from this study to eliminate its potential confounding effects on perioperative outcomes and complication rates, thereby ensuring a more direct comparison of the surgical techniques themselves. All enrolled patients provided written informed consent, agreeing to participate in the study and authorizing the use of their relevant data for research purposes [12].

### Surgical technique

All procedures were performed under general anesthesia by a single experienced surgeon. Prior to the first robotic case in this study, the surgeon had completed over 100 robotic-assisted urological procedures (including radical prostatectomy and partial nephrectomy) using the KangDuo surgical robot system, indicating that the initial learning curve for robotic platform handling had been overcome. Following cystectomy, all patients underwent extended lymph node dissection.

#### Patients in the control group underwent open ileal conduit diversion

Under laparoscopy, the left ureter was transposed to the right abdominal cavity through the natural space posterior to the sigmoid mesocolon without tension. A midline lower abdominal incision, approximately 5–6 cm in length, was made, and the abdominal cavity was entered layer by layer. The specimen bag was retrieved through the incision. At a point approximately 15–20 cm proximal to the ileocecal valve, a 15 cm segment of ileum with its vascular pedicle was isolated. The intestinal continuity was restored using a linear stapler-cutter, and the mesenteric defect was closed by suturing. The isolated ileal segment was repeatedly irrigated with saline until clean. The distal ends of the left and right ureters were each incised longitudinally for approximately 1.5 cm. The medial edges of the two ureters were approximated and sutured together using 5-0 absorbable sutures to create a single, larger common orifice (Wallace technique). The proximal end of the ileal conduit was anastomosed end-to-end to this common ureteral orifice using 5-0 absorbable sutures in a full-thickness, tension-free, mucosa-to-mucosa running suture. A single-J stent was inserted through the anastomosis proximally into the ureter, with its other end exteriorized through the ileal conduit.

#### Patients in the observation group underwent totally intracorporeal modified ileal conduit diversion

**Figure 2.**
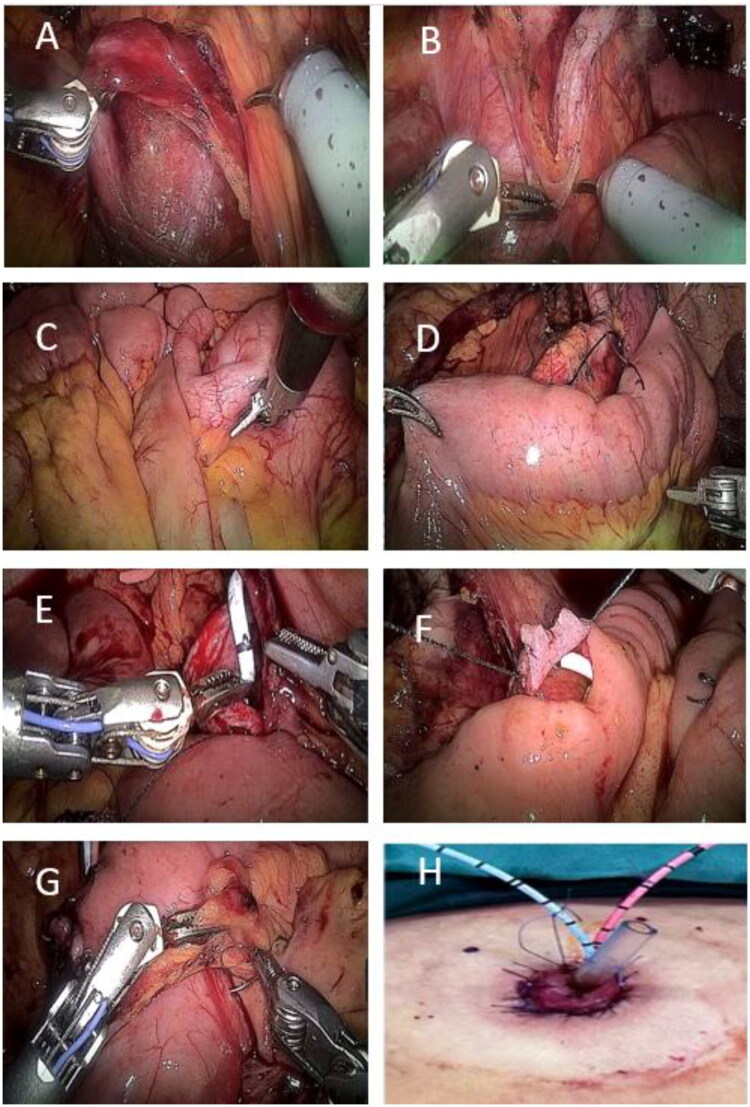
Surgical procedure of totally intracorporeal modified ileal conduit diversion. A surgical video demonstrating the key steps of this modified technique.

At the level of the ileocecal region, the right ureter was mobilized and transected, with care taken to preserve its adventitial blood supply. The distal end of the ureter was spatulated longitudinally for approximately 2 cm in preparation for anastomosis. On the sigmoid mesocolon, lateral to the inferior mesenteric artery, an avascular area was selected. A window was carefully created in this area, and the left ureter was identified and mobilized. The left ureter was then transposed into the abdominal cavity through this mesocolonic window. Its distal segment was transected, and the end was spatulated longitudinally for about 3 cm for anastomosis. Approximately 15–20 cm proximal to the ileocecal valve, the ileal mesentery was identified and dissected. A segment of ileum, approximately 20 cm in length, was isolated using an intracorporeal powered linear stapler cutter. Bowel continuity was restored using another linear stapler-cutter, and the mesenteric defect was closed by suturing. The proximal end of the isolated ileal segment was then brought anterior to the sigmoid mesocolon to the left side of the abdominal cavity. An end-to-end anastomosis between the proximal ileal end and the left ureter was performed using a 4-0 V-Loc barbed suture in a full-thickness, continuous fashion, ensuring good mucosal apposition and a tension-free anastomosis. On the right side, a small opening corresponding to the diameter of the distal right ureter was made on the antimesenteric border of the ileal conduit, approximately 5–8 cm distal to the left-sided anastomosis. An end-to-side anastomosis between the right ureter and the ileal conduit was performed using 4-0 V-Loc barbed suture. The space between the ileal conduit and the bowel was closed by suturing. During the anastomotic procedures, single-J stents were inserted through both ureteral anastomoses in a retrograde direction towards the renal pelvises (Figure 2).

At the preoperatively marked midpoint between the right anterior superior iliac spine and the umbilicus, a circular skin incision approximately 2–3 cm in diameter was made. The anterior rectus sheath was incised in a cruciate fashion, the rectus muscle was split longitudinally, and the peritoneum was opened with a cruciate incision. The distal end of the ileal conduit was delivered through this channel to the skin surface without torsion, forming a nipple approximately 2 cm in height. Several interrupted sutures were placed to secure the seromuscular layer of the bowel to the anterior rectus sheath. A “rosebud” technique was used to exert and suture the stoma. Both indwelling stents were then secured appropriately.

A. Exposure and mobilization of the left ureter through the mesocolonic window. B. Mobilization of the right ureter in the right paracolic gutter. C. Identification of the ileocecal junction. D. Selecting the ileal segment 20 cm from the ileocecal junction. E. Performing a ureteroileal end-to-end anastomosis in the left abdomen. F. Performing a ureteroileal end-to-side anastomosis in the right abdomen. G. Suture closure of the space between the ileal conduit and the bowel. H. Ileal stoma.

### Perioperative management

Since January 2022, we have implemented an Enhanced Recovery After Surgery (ERAS) protocol for all patients undergoing radical cystectomy. Compared with traditional rehabilitation care, ERAS is an evidence-based, multimodal, and multidisciplinary approach comprising a series of optimized measures. Its goal is to minimize surgical stress response and complications, thereby accelerating both physiological and psychological recovery in patients.

Preoperative functional exercises, including respiratory and physical training, and nutritional support were provided. Prolonged preoperative fasting was avoided. Gentle bowel preparation was performed with oral laxatives the night before surgery. Fasting was initiated 6 h before anesthesia, and clear fluid intake was stopped 2 h preoperatively. Clear carbohydrate-rich drinks were administered 10 h and 2 h before surgery to alleviate insulin resistance, anxiety, and thirst. Perioperative prophylactic antibiotics were routinely administered for 3 days, with extended use in cases of fever or elevated infection markers. Postoperative analgesia was managed using continuous epidural analgesia combined with oral analgesics. Early ambulation was encouraged, and patients were assisted by family members to get out of bed as early as the first postoperative day. Oral intake was initiated with clear liquids on postoperative day 1, gradually advancing to a full liquid and then a soft diet based on tolerance. Nasogastric tubes were not routinely placed. Drains were removed if the output remained below 50 ml/day for two consecutive days. Ureteral stents were removed one month postoperatively, unless persistent symptoms such as flank pain or fever necessitated reinsertion. A stoma therapist provided comprehensive management throughout the process, guiding patients and their families in stoma care. This included monitoring stoma blood supply and color, as well as preventing complications such as peristomal dermatitis, stenosis, and retraction.

#### Discharge criteria

Patients were considered eligible for discharge when they met the following criteria: stable vital signs, ability to tolerate oral intake, adequate pain control with oral analgesics, ability to ambulate independently or with minimal assistance, and acquisition of basic stoma care skills.

Patients were followed up consecutively for 1 month postoperatively, then every 3 months during the first year, every 6 months during the second year, and annually thereafter. Routine follow-up assessments included physical examination and serological tests, such as complete blood count, electrolyte panel, serum creatinine, and liver function tests. Chest computed tomography (CT) and abdominal/pelvic CT were performed at 6 months after cystectomy and subsequently once yearly for 5 years or as clinically indicated.

### Data collection

This study employed a retrospective research design. Patient clinical data were systematically collected by reviewing our institution’s electronic medical record system and electronic databases. The collected data primarily included: Baseline Data: Patient demographic characteristics (age, sex, body mass index), preoperative comorbidities (Charlson Comorbidity Index), American Society of Anesthesiologists (ASA) physical status classification, performance status, tumor-related characteristics (tumor size, clinical and pathological T stage, tumor grade, number of tumors), and the presence or absence of preoperative upper urinary tract hydronephrosis. Perioperative Data: Operative time, intraoperative blood loss, indicators of postoperative bowel function recovery (time to return of bowel sounds, time to first flatus, time to first defecation), duration of drain placement, postoperative hospital stay, and time to ureteral stent removal. Postoperative Complication Data: Complications occurring within 90 days postoperatively were recorded, with a focus on the incidence of fever, ileus, and ileal stoma obstruction. The occurrence of newly developed upper urinary tract calculi and ureteroileal anastomotic stricture during the follow-up period was also documented. Ureteroileal anastomotic stricture was diagnosed and confirmed by postoperative contrast-enhanced CT urography (CTU), defined as a lumen diameter <2 mm at the anastomotic site accompanied by ipsilateral ureteral and renal pelvic dilatation (anterior-posterior renal pelvic diameter >2 cm). Furthermore, all patients diagnosed with stricture in this study underwent ureteroscopic confirmation and concurrent balloon dilation, ensuring the objectivity and accuracy of the endpoint assessment. The median follow-up time for patients was recorded.

The study duration spanned from January 2023 to October 2025 for patient enrollment and data collection. The data analysis was completed in December 2025.

### Data analysis

All data were processed and analyzed using SPSS statistical software. Continuous variables were first assessed for normality. Those conforming to a normal distribution are presented as mean ± standard deviation, and comparisons between groups were performed using the independent samples *t* test. Data not conforming to a normal distribution are presented as median (interquartile range), with group comparisons made using the Mann–Whitney *U* test. Categorical variables are expressed as number (percentage), and group comparisons were conducted using the Chi-square test or Fisher’s exact test (when the expected frequency was <5). A *p* value <0.05 was considered statistically significant. A *p* value <0.05 was considered statistically significant. Post-hoc power analysis was performed for the primary endpoint using G*Power software (version 3.1). Based on the sample sizes (*n* = 23 and *n* = 46) and the observed stricture rates (0% vs. 15.22%), the achieved power was calculated as 68.4%. To adjust for potential confounders, multivariable logistic regression analysis was performed for the primary endpoint (ureteroileal anastomotic stricture). Due to the zero-event occurrence in the robotic group, Firth’s penalized likelihood logistic regression was applied, which is specifically designed for small-sample and rare-event data. Covariates included in the model were clinical T stage (due to its imbalance between groups, *p* = 0.046), age, and preoperative hydronephrosis (as a clinically relevant factor for ureteral stricture). Results are presented as adjusted odds ratios (OR) with 95% confidence intervals (CI).

## Results

### Comparison of baseline patient characteristics

A total of 69 patients were ultimately included in this study: 23 in the robot-assisted totally intracorporeal surgery group (Robot-assisted Group) and 46 in the traditional laparoscopic combined with open ileal conduit surgery group (Laparoscopic Group). As shown in Table 1, there were no statistically significant differences (*p* > 0.05) between the two groups in baseline characteristics including age, sex, body mass index, Charlson Comorbidity Index, ASA classification, ECOG performance status, tumor size, pathological T stage, tumor grade, number of tumors, preoperative upper urinary tract hydronephrosis, and lymph node status, indicating comparability. The only difference was observed in the distribution of clinical T stage (*p* = 0.046), with a higher proportion of T1 stage patients in the Robot-assisted Group (26.09% vs. 10.87%) and a higher proportion of T2 stage patients in the Laparoscopic Group (80.43% vs. 60.87%).

**Table 1.** Comparison of baseline characteristics between the two groups[(mean ± SD), *n*(%)].

Variable	Robot-assisted group(*n* = 23)	Laparoscopic group(*n* = 46)	*p* 值
**Age (years)**	72.16 ± 3.35	71.99 ± 4.44	0.253
**Sex**			0.246
Male, *n* (%)	16(69.57%)	37(80.43%)	
Female, *n* (%)	7(30.43%)	9(19.57%)	
BMI(kg/m^2^)	24.21 ± 1.75	24.01 ± 2.29	0.064
Charlson Comorbidity Index, *n* (%)			0.920
≤2	9(39.13%)	16(34.78%)	
>2	14(60.87%)	30(65.22%)	
ECOG performance status, *n* (%)			0.640
0	14(60.87%)	26(56.52%)	
1	9(39.13%)	20(43.48%)	
**ASA classification, *n* (%)**			0.820
1	6(26.09%)	12(26.09%)	
2	17(73.91)	34(73.91%)	
Mean tumor size (cm)	4.65 ± 0.61	4.50 ± 0.73	0.111
Clinical *T* stage, *n* (%)			0.046
T1	6(26.09%)	5(10.87%)	
T2	14(60.87%)	37(80.43%)	
T3	3(13.04%)	4(8.7%)	
**Pathological *T* stage, *n* (%)**			0.110
T1	2(8.69%)	4(8.69%)	
T2	16(69.56%)	35(76.09%)	
T3	5(21.75%)	7(15.22%)	
Tumor grade, *n* (%)			0.620
G1	3(13.04%)	3(6.52%)	
G3	20(86.96%)	43(93.48%)	
**Number of tumors**	2.35 ± 0.65	2.13 ± 1.18	0.198
**Presence of upper urinary tract Hydronephrosis, *n* (%)**			0.840
Yes	11(47.83%)	20(43.48%)	
No	12(52.17%)	26(56.52%)	
Lymph node status, *n* (%)			0.344
Positive	3(13.04%)	11(23.91%)	
Negative	20(86.96%)	35(76.09%)	

BMI: Body mass index; ECOG: Eastern Cooperative Oncology Group; ASA: American Society of Anesthesiologists.

### Comparison of perioperative outcomes

Regarding perioperative parameters (Table 2), the median operative time was longer in the robotic group than in the laparoscopic group [(249.69 ± 19.71) min vs. (199.62 ± 22.65) min, *p* < 0.001]. However, intraoperative blood loss was significantly less in the robotic group compared with the laparoscopic group [(98.70 ± 28.97) mL vs. (155.00 ± 65.76) mL, *p* < 0.001].

**Table 2.** Comparison of perioperative data between the two groups [(mean ± SD), *n* (%)].

Indicator	Robot-assisted group(*n* = 23)	Laparoscopic group(*n* = 46)	*p* 值
Operative time(min)	249.69 ± 19.71	199.62 ± 22.65	<0.001
Intraoperative blood loss(ml)	98.70 ± 28.97	155.00 ± 65.76	<0.001
Time to bowel sound recovery(h)	17.58 ± 2.89	17.47 ± 2.96	0.596
**Time to first flatus(h)**	30.73 ± 3.24	30.54 ± 3.38	0.268
Time to first defecation(h)	51.55 ± 6.78	51.25 ± 6.40	0.257
**Time to drain removal(d)**	6.29 ± 1.03	7.79 ± 0.77	<0.001
Postoperative hospital stay(d)	8.33 ± 0.83	9.72 ± 0.87	<0.001
Time to ureteral stent removal(d)	26.18 ± 3.85	32.14 ± 2.97	<0.001
Serum creatinine(μmol/L)			
Preoperative	94.82 ± 11.72	96.93 ± 12.83	
Postoperative 6 months	96.58 ± 11.95	121.45 ± 13.27	
Change value	1.76 ± 2.87	24.52 ± 3.95	<0.001

Regarding postoperative recovery indicators, there were no significant differences between the two groups in the time to recovery of bowel function (including time to bowel sound recovery, first flatus, and first defecation) (all *p* > 0.05). However, the time to drain removal [(6.29 ± 1.03) d vs. (7.79 ± 0.77) d, *p* < 0.001], postoperative hospital stay [(8.33 ± 0.83) d vs. (9.72 ± 0.87) d, *p* < 0.001], and time to ureteral stent removal [(26.18 ± 3.85) d vs. (32.14 ± 2.97) d, *p* < 0.001] were all significantly shorter in the robotic group compared to the laparoscopic group. At 6 months postoperatively, the increase in serum creatinine levels was significantly lower in the robotic group than in the laparoscopic group [(1.76 ± 2.87) μmol/L vs. (24.52 ± 3.95) μmol/L, *p* < 0.001].

### Postoperative complications and follow-up

Regarding the primary endpoint, the incidence of ureteroileal anastomotic stricture was significantly lower in the robotic group compared to the laparoscopic group [0.0% (0/23) vs. 15.22% (7/46), *p* = 0.043].

For secondary endpoints, the robotic group also demonstrated significantly lower rates of postoperative intestinal obstruction [8.70% (2/23) vs. 28.26% (13/46), *p* = 0.043] and newly developed upper urinary tract stones [4.35% (1/23) vs. 21.74% (10/46), *p* = 0.046]. There were no significant differences between the two groups in the incidence of postoperative fever or ileal stoma obstruction (all *p* > 0.05).

All patients completed follow-up for more than 6 months. Due to the different initiation times of the surgical procedures, the median follow-up duration was significantly shorter in the robotic group compared to the laparoscopic group [12 (9–14) months vs. 24 (20–28) months, *p* < 0.001] (Table 3). Importantly, in the laparoscopic group, all 7 ureteroileal anastomotic strictures occurred within the first 6 months postoperatively (median time to stricture diagnosis: 4.2 months, range 3.1–5.8 months). Therefore, given that the robotic group had a follow-up duration exceeding 6 months, we consider the two groups comparable for the primary endpoint of anastomotic stricture. Nevertheless, longer-term follow-up of the robotic cohort is ongoing to monitor for any late-onset complications.

**Table 3.** Comparison of postoperative complications between the two groups [(mean ± SD), *n* (%)].

Indicator	Robot-assisted group (*n* = 23)	Laparoscopic group (*n* = 46)	*p* 值
Fever, *n* (%)	15 (65.22%)	32 (69.57%)	0.705
Intestinal obstruction, *n* (%)	2 (8.70%)	13 (28.26%)	0.043
Newly developed upper urinary tract stones, *n* (%)	1 (4.35%)	10 (21.74%)	0.046
Ureteroileal anastomotic stricture, *n* (%)	0 (0%)	7 (15.22%)	0.043
Ileal stoma obstruction, *n* (%)	4 (17.39%)	8 (17.39%)	1.000
Median follow-up time (*m*)	12 (9–14)	24 (20–28)	<0.001

### Multivariable analysis

To adjust for the imbalance in clinical T stage between the two groups (*p* = 0.046) and other potential confounders, multivariable Firth logistic regression was performed for the primary endpoint of ureteroileal anastomotic stricture. After adjusting for clinical T stage, age, and preoperative hydronephrosis, the robotic-assisted modified technique remained independently associated with a significantly lower risk of anastomotic stricture compared to the laparoscopic approach (adjusted OR = 0.11, 95% CI: 0.01–0.87, *p* = 0.035). The full results of the multivariable analysis are presented in Table 4.

**Table 4.** Multivariable firth logistic regression analysis for ureteroileal anastomotic stricture.

Variable	Adjusted OR	95% CI	*p* value
Robotic-assisted modified technique (vs. laparoscopic)	0.11	0.01–0.87	0.035
Clinical T stage (T2/T3 vs. T1)	1.52	0.32–7.21	0.602
Age (per 1-year increase)	1.03	0.92–1.15	0.589
Preoperative hydronephrosis (yes vs. no)	1.85	0.41–8.33	0.423

## Discussion

This study systematically compares robot-assisted totally intracorporeal modified ileal conduit urinary diversion with conventional laparoscopic combined with open ileal conduit urinary diversion, comprehensively evaluating the perioperative safety and long-term efficacy of this novel surgical approach. Our findings not only confirm the inherent advantages of robotic surgery in terms of minimally invasive techniques [13], but more importantly, reveal significant benefits of the robot-assisted modified ileal conduit procedure in reducing key long-term complications. These benefits are primarily attributed to its technical precision and the improved surgical design [14].

The two groups were comparable in terms of age, gender, comorbidities, performance status, and most oncological characteristics, ensuring the reliability of subsequent outcome comparisons. The only difference was observed in the distribution of clinical T-stage. While some studies suggest that tumor stage is an independent prognostic factor for patients [15], the primary assessment in this study is based on postoperative pathological staging. Both groups exhibited consistent pathological staging, and the preoperative clinical staging did not influence surgical selection or decision-making. Therefore, it does not affect the analysis of postoperative data. Regarding surgical feasibility, the significantly longer operative time in the robotic group reflects the technical challenges associated with performing complete intracorporeal cystectomy, lymph node dissection, and complex urinary diversion. This finding is consistent with studies by international scholars, which indicate that robotic surgery tends to have longer operative times compared to open procedures [16]. However, the significantly reduced intraoperative blood loss demonstrates the advantages of the robotic system in precise dissection and vascular control [17], establishing a solid foundation for subsequent patient recovery.

Regarding postoperative recovery indicators, the results demonstrated a notable “dissociation” phenomenon. Although no differences were observed between the two groups in the time to recovery of bowel function—including bowel sounds, flatus, and defecation—this may be attributed to the dominant role of a unified Enhanced Recovery After Surgery (ERAS) protocol, highlighting the importance of standardized pathways as emphasized in the latest ERAS guidelines. However, the robotic group showed significantly shorter times to drain removal, postoperative hospital stay, and ureteral stent removal. These findings clearly reflect the advantages of the robot-assisted modified technique, which, through minimized surgical trauma, reduced postoperative exudation, and optimized drainage, facilitates earlier removal of both external drains and internal stents. This ultimately translates into a shorter hospital stay, aligning with recent studies reporting that robotic surgery promotes accelerated postoperative recovery [18].

The most significant finding of this study lies in the preventive effect of the robot-assisted modified technique against major long-term complications. The incidence of ureteral anastomotic stricture was 0% in the robotic group, significantly lower than the 15.22% observed in the laparoscopic group, contrasting sharply with the reported incidence of anastomotic stricture associated with traditional techniques in the literature [19]. This achievement stems from multiple mechanistic refinements. First, the modified technique involves pulling the ileal segment anterior to the colon to the left side for anastomosis with the left ureter, avoiding the long-distance mobilization and potential devascularization or twisting of the left ureter across the spine required in traditional approaches [3]. This fundamentally reduces the risk of ischemic stricture. Second, the robotic system provides a three-dimensional magnified view and wristed instruments, enabling surgeons to precisely spatulate the distal ureter and perform continuous suturing to achieve a wide, tension-free, and mucosa-to-mucosa aligned anastomosis. This precision far surpasses that achievable through a small incision for extracorporeal manipulation, fully demonstrating the advantages of robotic surgery for reconstructive procedures [20]. Finally, creating a relatively large anastomotic orifice (3 cm on the left, 2 cm on the right) is also crucial for stricture prevention. While concerns existed that a larger orifice might lead to significant reflux, the antegrade peristalsis of the intestinal segment has alleviated this concern, as confirmed by the mature experience with intestinal ureter replacement surgery at Peking University First Hospital [21]. Furthermore, the stable postoperative serum creatinine levels have dispelled our initial apprehensions. Although the extracorporeally performed Wallace technique creates a common channel, it is inferior to fully intracorporeal robotic anastomosis in terms of visualization, operative precision, and minimizing tissue traction injury. Studies by other scholars using the Wallace technique also report a certain incidence of anastomotic stricture [22]. To facilitate reproducibility, we have provided a detailed surgical video (Supplementary Video 1) and a step-by-step illustration (Figure 2) of the modified antecolic anastomosis technique.

The incidence of postoperative intestinal obstruction was significantly lower in the robotic group (8.7%) compared to the laparoscopic group (28.26%), which can be attributed to dual preventive mechanisms at both anatomical and functional levels. Anatomically, the traditional Wallace technique for anastomosis creates potential spaces between the juxtaposed ureters and between the ureters and the bowel segment, which are prone to internal herniation leading to intestinal obstruction. In contrast, the modified ileal conduit technique completely eliminates these spaces, fundamentally preventing internal hernia formation and thereby reducing the incidence of intestinal obstruction [23]. Additionally, the totally intracorporeal approach avoids the need for a larger auxiliary incision; in female patients, specimen extraction *via* the vaginal route eliminates the auxiliary incision altogether. This reduces postoperative pain and inflammatory responses, promotes earlier ambulation, and collectively mitigates the risk of paralytic ileus [24]. The incidence of newly developed upper urinary tract stones was markedly lower in the robotic group (4.35%) than in the laparoscopic group (21.74%). A patent, non-strictured anastomosis along with a naturally aligned ureteral course ensures effective drainage of the upper urinary tract, prevents urinary stasis, and thereby eliminates a nidus for stone formation [25].

There was no significant difference between the two groups in the incidence of postoperative fever and ileal stoma obstruction. Postoperative fever is often associated with infection or absorption fever, and the standardized perioperative antibiotic protocol likely mitigated any potential influence of surgical approach, as studies have shown that standardized management can reduce the impact of procedural variations on certain outcomes [26]. Stoma obstruction is more closely related to the technique of constructing the abdominal wall channel and postoperative edema, which have less association with the choice of intra-abdominal surgical technique. Furthermore, all patients who experienced stoma obstruction showed improvement after dilation of the stoma channel.

Acknowledging the potential confounding effect of the surgeon’s learning curve, the senior author had accumulated substantial robotic experience prior to this study. Nevertheless, the temporal sequence (robotic cases performed later) means that improvements could partly reflect surgical maturation rather than the technical modification alone. Furthermore, the control group underwent extracorporeal ureteroileal anastomosis through a small incision, which—although not laparoscopic—still offers inferior visualization and suturing precision compared to robotic intracorporeal anastomosis. Therefore, the observed differences in stricture rates may be attributable to a combination of the robotic platform, the totally intracorporeal approach, and the modified antecolic routing. Direct comparison between robotic intracorporeal traditional retrocolic versus robotic intracorporeal modified antecolic techniques is needed to isolate the specific benefit of the anatomical modification.

Although this study observed significant benefits of robot-assisted combined with modified ileal conduit surgery for patients, several limitations remain. First, this is a single-center, non-randomized retrospective study, which may be subject to selection bias. Second, and most critically, due to the later adoption of robotic technology at our center, there is a significant difference in the median follow-up time between the two groups. We acknowledge that this temporal bias may lead to underestimation of late-onset complications in the robotic group. However, since all ureteral anastomotic strictures in the control group occurred within 6 months postoperatively and the robotic group was followed for over 6 months, we consider the results comparable for the primary endpoint. Nonetheless, longer-term follow-up of the robotic cohort is ongoing to monitor for any delayed strictures or other complications. Third, the disparity in follow-up duration prevented us from comparing oncological outcomes between the two groups, and longer-term follow-up is still required. Fourth, all surgeries were performed by the same experienced surgeon, and the reproducibility of these results during broader technical dissemination needs validation across multiple centers. Nevertheless, multivariable Firth logistic regression confirmed that the robotic-assisted modified technique remained an independent protective factor against anastomotic stricture after adjusting for clinical T stage and other confounders (adjusted OR = 0.11, 95% CI: 0.01–0.87, *p* = 0.035). Finally, the small sample size and the zero-event nature of the primary outcome in the robotic group make the statistical significance fragile; post hoc power analysis showed a power of 68.4%, below the conventional 80% threshold. Therefore, our findings should be considered exploratory and require confirmation in larger prospective studies.

In summary, robot-assisted totally intracorporeal radical cystectomy with modified ileal conduit urinary diversion not only ensures perioperative safety but also demonstrates substantial potential in comprehensively accelerating postoperative recovery. Notably, through its technical precision and modified anatomical approach, this technique fundamentally reduces the risk of key long-term complications such as ureteroileal anastomotic stricture, postoperative intestinal obstruction, and upper urinary tract stones. Despite its limitations, the significant benefits it offers for patients’ long-term quality of life and preservation of upper urinary tract function establish it as a highly promising and superior surgical option. Future prospective randomized controlled trials with extended follow-up periods are warranted to further substantiate the conclusions of this study.

## Data Availability

The surgical video supporting this study is openly available in Science Data Bank at https://doi.org/10.57760/sciencedb.38790 (DOI: 10.57760/sciencedb.38790; CSTR: 31253.11.sciencedb.38790). All other original contributions presented in the study are included in the article and its Supplementary Material. Further inquiries can be directed to the corresponding author.
